# Hepatitis B virus promotes hepatocellular carcinoma development by activating GP73 to repress the innate immune response

**DOI:** 10.1186/s13027-022-00462-y

**Published:** 2022-10-04

**Authors:** Long Liu, Yanping Huang, Yanan Fu, Jingjing Rao, Feng Zeng, Manshan Ji, Xiang Xu, Jianyong Zhu, Weixing Du, Zhixin Liu

**Affiliations:** 1 Department of Infectious Diseases, Department of Respiratory, Renmin Hospital, Hubei University of Medicine, Shiyan, China; 2 School of Basic Medical Sciences, Hubei University of Medicine, Shiyan, China; 3 Institution of Virology, Hubei University of Medicine, Shiyan, China

**Keywords:** Hepatitis B virus, Hepatocellular carcinoma, Golgi Protein 73

## Abstract

**Background:**

Hepatitis B virus (HBV) causes acute and chronic infection in the clinic. Hepatocellular carcinoma (HCC) is closely linked to HBV infection. Serum Golgi protein 73 (GP73) increases during HBV infection. However, the role of GP73 during HBV infection and the occurrence of HBV-related HCC is still poorly understood.

**Methods:**

The underlying role of HBV-induced GP73 in regulating HCC development was investigated in this study. GP73 expression in HBV-related clinical HCC tissues and in HBV-infected hepatoma cells and primary human hepatocytes was evaluated by immunohistochemistry, ELISAs, Western blotting and quantitative real-time PCR (qRT-PCR) analysis. Tumorigenicity of GP73 overexpressed cells was detected by flow cytometry, qRT-PCR, xenograft nude mouse analyses and sphere formation assays. The effects of GP73 and HBV infection on host innate immune responses in hepatocytes were further investigated by Western blotting and qRT-PCR analysis.

**Results:**

Initially, we confirmed that HBV-positive HCC tissues had significantly higher expression of GP73. Ectopic expression of the HBV gene could induce GP73 expression in primary human hepatocytes and hepatoma cells in vitro. In addition, we discovered that GP73 promotes HCC in both normal liver cells and hepatoma cells. We also found that ectopic expression of HBV genes increases GP73 expression, suppressing the host's innate immune responses in hepatocytes.

**Conclusions:**

Our results demonstrate that HBV facilitates HCC development by activating GP73 to repress the host's innate immune response. This study adds to our understanding of the pathogenesis of HBV infection-induced HCC. The findings also provide preclinical support for GP73 as a potential HCC prevention or treatment target.

**Supplementary Information:**

The online version contains supplementary material available at 10.1186/s13027-022-00462-y.

## Introduction

Hepatitis B virus (HBV) causes acute and chronic infection in the clinic [1]. More than 2 billion people worldwide have been infected with HBV [2]. Approximately 5–10% of adults develop into chronic carriers after acute HBV infection, and up to 30% of chronic carriers eventually develop hepatitis, fibrosis, or cirrhosis, resulting in hepatocellular carcinoma (HCC) [3]. Chronic HBV infection can easily lead to the occurrence of HCC [4, 5], but the molecular mechanism of HBV infection-induced HCC is still not fully understood.

During HBV infection, hepatocytes begin to abnormally express some proteins, among which GP73 (Golgi protein 73) shows upregulated expression [6]. GP73 is a Golgi protein identified by Kladney et al*.* [7] in a study of adult giant cell hepatitis (GCH). GP73 is rarely expressed in hepatocytes in normal liver tissues and is mainly found in bile duct epithelial cells in the portal area [7]. Clinical evidence has shown that the concentration of serum GP73 is significantly higher in patients with hepatocyte damage caused by viral invasion [8]. The physiological functions of GP73 is a TBC-domain Rab GTPase-activating protein regulating ApoB export and reshapes the lipid metabolism of hepatocytes in patients with acute or chronic liver diseases [9]. However, most studies focus on the relationship between abnormal expression of GP73 and liver carcinogenesis [10–12], and the role of GP73 during HBV infection is poorly understood. Here, we identified that GP73 promotes HCC development by repressing the host's innate immune response. We found that HBV-positive HCC tissues had higher GP73 levels than HBV-negative HCC tissues. The forced expression of HBV genes activates GP73 in primary human hepatocytes (PHHs) and hepatoma cells. More importantly, genetic overexpression of GP73 promotes the appearance of liver cancer stem cells (CSCs) and enhanced HepG2 and Huh7 cell tumorigenesis in nude mice. We further found that GP73 repressed the expression of NK-κB, IFN-λ1, IFN-β, TNF-α, and IL-6. These results indicated that HBV facilitates HCC development by activating GP73 to repress the host's innate immune response.

## Materials and methods

### Clinical samples and immunohistochemistry

Formalin-fixed and paraffin-embedded liver cancer tissues were obtained from patients undergoing surgery at Renmin Hospital, Hubei University of Medicine, ShiYan, China. Six HBsAg-positive HCC tissues and six HBsAg-negative HCC tissues were selected for analysis. The selected pathological tissues were negative for hepatitis A, C, D, and E viruses and negative for human immunodeficiency virus (HIV). Immunohistochemical staining and Western blot analysis were performed as previously described [13]. The antibody used for staining was anti-GP73 (#ab109628, Abcam). The study was conducted according to the principles of the Helsinki Declaration and approved by the Institutional Review Committee of Hubei Medical University according to its guidelines for protecting human subjects. Each participant provided written informed consent.

### Xenograft nude mice

Male BALB/cA-nu mice (19.3–23.6 g) aged 5 weeks (n = 6 in each group) were purchased from Beijing HFK Bioscience Co., Ltd. (Beijing, China). The tumorigenicity assay in nude mice was performed as described previously [13]. Animal conservation and sacrifice were carried out according to the methods approved by the Animal Care and Use Committee of the Animal Experimental Center of Hubei Medical University.

### Cell culture and transfection

Primary human hepatocytes (PHHs) were purchased from the Research Institute for Liver Diseases (Shanghai, China) and cultured as described previously [14]. HepG2 cells, Huh-7 cells, HepG2.2.15 cells, and HEK293T cells were purchased from the China Center for Type Culture Collection (CCTCC, Wuhan, China). L02 cells and HepG2-NTCP cells were kindly provided by Dr. Jianguo Wu of Wuhan University, China. HepG2 cells, HepG2-NTCP cells, HepG2.2.15 cells, Huh-7 cells, L02 cells, and HEK293T cells were cultured as previously reported [15]. For PHHs, the plasmid was transfected into cells with an Amaxa Mouse/Rat Hepatocyte Nucleofector™ Kit (Lonza, Basel, Switzerland) following the optimized protocol from the manufacturer's instructions. For other cells, Lipofectamine® 3000 (Thermo Fisher Scientific, USA) was employed to transfect cells with plasmid or small interfering RNAs according to the manufacturer's instructions.

### Plasmid construction

For overexpressing GP73, specific primers were used to amplify the coding region of *GP73* by PCR (forward: 5′-GCG GAA TTC ATG ATG GGC TTG GGA AAC-3′; reverse: 5′-CCG CTC GAG TCA GAG TGT ATG ATT CCG-3′). The *GP73* PCR product was inserted into the pCMV-Tag2B or pWPXLD vector. For knockdown of GP73, the following sequences were used to target the human GP73 CDS: 5′-GTTGAGAAAGAGGAAACCAAT-3′ [16].

All constructs were confirmed by DNA sequencing.

### Virus production and transduction of cell lines

For HBV infection of HepG2-NTCP cells, the culture supernatant of the HepG2.2.15 cell line was concentrated 100-fold by ultracentrifugation. The concentrated HBV stock titer (genome equivalents/ml, GEq/ml) was assessed using qRT-PCR. The multiplicity of infection (MOI) was defined as the genome equivalents per cell (GEq/cell). HepG2-NTCP cells were infected as described previously [15].

Construction of the stable GP73 knockdown cell line was performed as previously described [13]. Briefly, the lentivirus plasmid pWPXLD (Addgene plasmid #12258) was modified by inserting a T2A peptide between the multiple cloning site (MCS) and the GFP gene. Then, the *gp73* gene was amplified by PCR and inserted into the MCS of pWPXLD. After that, lentivirus was produced in HEK293T cells and transfected into Huh7 cells or HepG2 cells. The GFP-positive cells were then enriched by flow cytometric sorting on the basis of GFP expression and named Huh7-GP73 or HepG2-GP73.

Construction of the stable GP73 knockdown cell line was performed as previously described [16]. Briefly, short hairpin sequences targeting *gp73* were inserted into the pLKO.1-TRC vector (Addgene #10879). After that, lentivirus was produced in HEK293T cells. Concentrated shRNA lentiviruses were used to transfect Huh7-GP73 cells or HepG2-GP73 cells. Transfected cells were cultured in puromycin (2.5 μg/ml) selection medium for at least 7 days to establish stable GP73 knockdown cell lines, which were named Huh7-GP73-RNAi or HepG2-GP73-RNAi.

### ELISA, Western blotting and immunofluorescence

For ELISAs, cell culture supernatants were collected to detect the levels of HBeAg and HBsAg with an ELISA kit (Kehua Bioengineering, Shanghai, China).

Western blot analysis was performed as described previously [13]. Antibodies were used to detect HA tags (#SAB2702196, Sigma), FLAG tags (#F7425, Sigma), GP73 (#ab109628, Abcam), NF-κB (p50) (#ab7549, Abcam), IFN-β (#ab180616, Abcam), IFN-λ1 (# MA5-30682, Invitrogen), IL-6 (#ab9324, Abcam), TNF-α (#ab6671, Abcam) and β-actin (#A1978, Sigma). ImageJ (http://rsb.info.nih.gov/ij/) software was employed for band intensity quantification of the Western blot results.

### Quantitative RT-PCR analysis

Total RNA was extracted from cells using TRIzol reagent (Invitrogen). RT-PCR primers were shown in Additional file 1: Table S1. Expression level data were normalized to the GAPDH expression level in each sample.

HBV DNA was detected by TaqMan real-time PCR using the following primers: 5′-AGA AAC AAC ACA TAG CGC CTC AT-3′, 5′- TGC CCC ATG CTG TAG ATC TTG-3′ and probe 5′-TGT GGG TCA CCA TAT TCT TGG G-3′.

### Flow cytometry

A CytoFlex (Beckman Coulter, USA) or MoFlo flow cytometer (Beckman Coulter, USA) was employed to analyze GFP signals or cell immunophenotyping. Single-cell suspensions were tested by cell immunophenotyping analysis after cells were stained with fluorescently labeled antibodies: CD90-APC (clone 5E10, eBioscience, 1:100), CD133-APC (clone AC133, Miltenyi Biotec, 1:100), and CD117-APC (clone 104D2, eBioscience, 1:100). Flow cytometry data were analyzed using CytExpert software (Beckman Coulter, USA).

### Wound healing assay

Cells were plated in 6-well plates. When the cells grew to 80%-90% confluence, the cell monolayers were scraped with a sterile micropipette tip. In the next step, wounded monolayers were gently washed with phosphate buffer solution (PBS) to remove cell debris. At three time points (0, 24 and 48 h), the distance between the two edges of the wound was calculated for three different positions.

### Sphere formation assay

Sphere formation assays were performed as previously reported [13]. HepG2-GFP cells and HepG2-GP73 cells were plated in ultralow attachment 6-well plates (Corning, Inc., Corning, NY, USA) at a density of 2000 cells/well. For Huh7-GFP and Huh7-GP73, a single cell was successively sorted into ultralow attachment 96-well plates by a MoFlo flow cytometer (Beckman Coulter, USA).

### Statistical analysis

All experiments were performed in triplicate. All data were recorded as the means ± standard deviations (SD) unless otherwise stated. Prism 5 software (GraphPad Software) was used for statistical tests. *P* < 0.05 was considered statistically significant.

## Results

### HBV induces GP73 expression in clinical HCC tissues

**Fig. 1:**
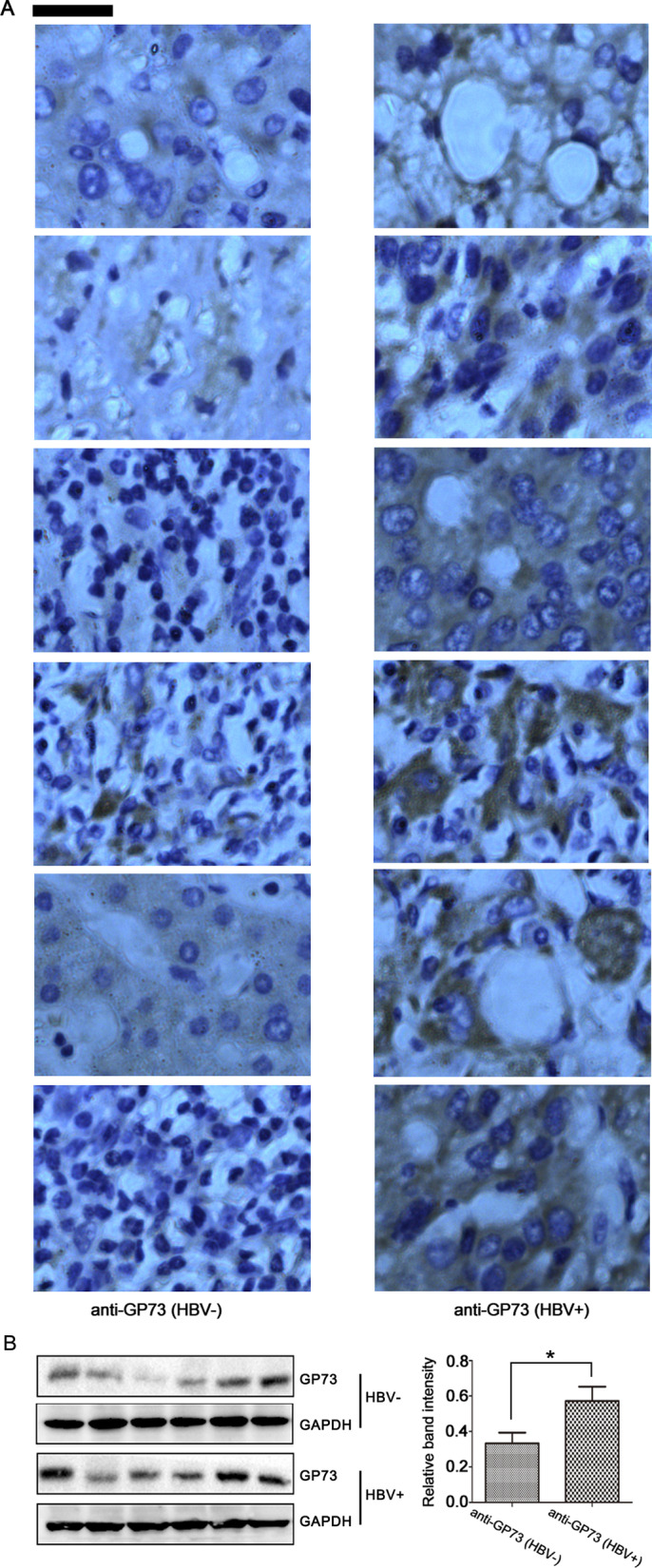
HBV infection facilitates GP73 expression in HCC tissues. **A** Immunohistochemical staining of GP73 in HCC samples (scale bar = 200 μm). **B** Western blot analysis of GP73 in HCC samples (left). Western blot band intensity was quantified using ImageJ software (right). The graphs show the means ± SDs, n = 6. *P < 0.05 compared with the control group

It has been reported that viral invasion can induce the expression of GP73 in hepatocytes [17]. The concentration of serum GP73 is significantly increased in patients with liver injury caused by virus infection [8]. Human carcinoma tissues were analyzed to validate the role of GP73 in HBV-related HCC. Immunohistochemistry analysis showed that the expression of GP73 was higher in HBV^−^positive HCC tissues than in HBV-negative HCC tissues (Fig. 1A). Western blot analysis also showed that HBV-positive HCC tissues had higher GP73 expression levels than HBV-negative HCC tissues (Fig. 1B). These results suggest that HBV infection induces GP73 expression in HCC tissues.

### HBV induces the expression of GP73 in hepatoma cells and primary human hepatocytes

**Fig. 2:**
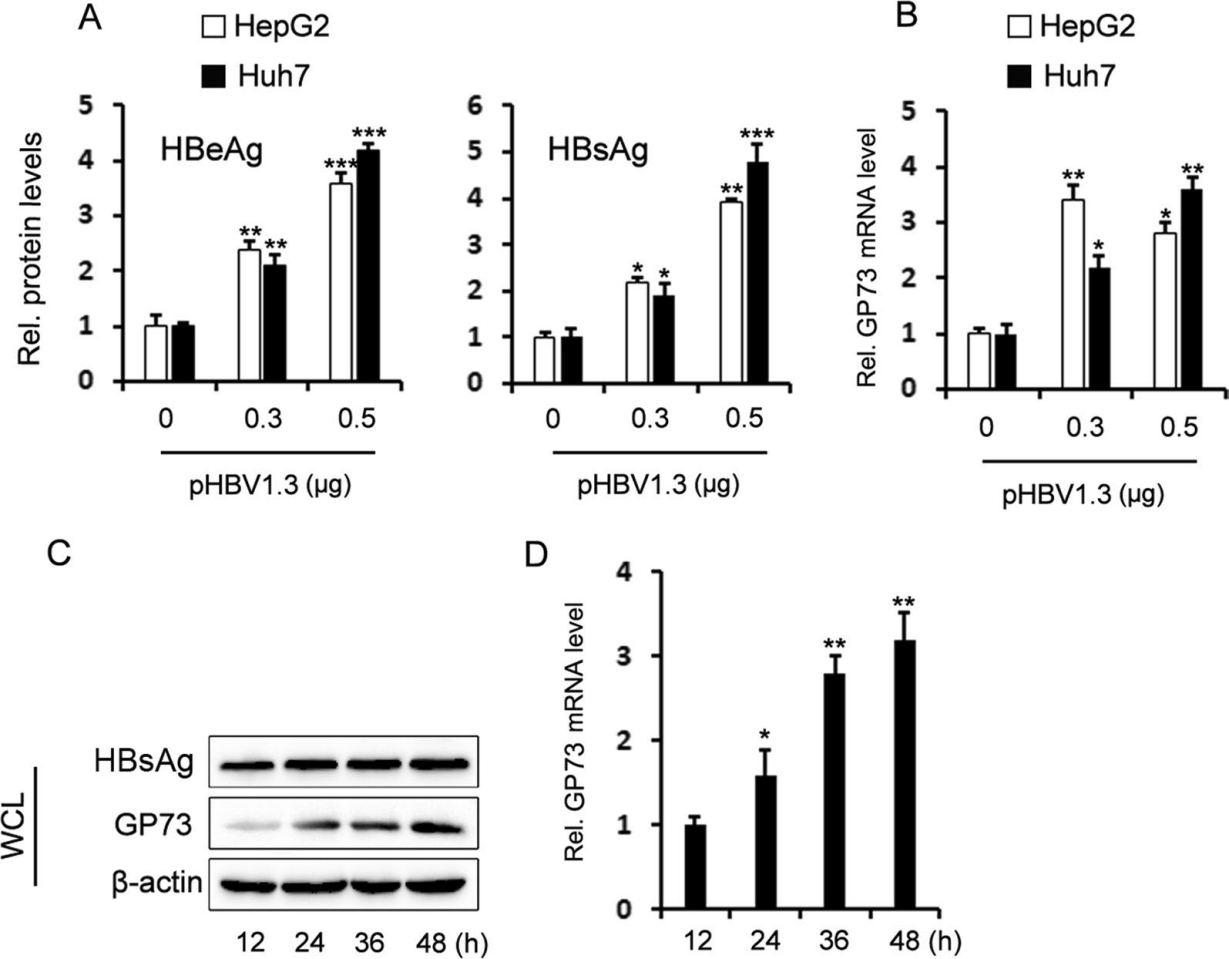
HBV induces activation of GP73. **A** pHBV1.3 at different doses was transfected into HepG2 and Huh7 cells. After 48 h, HBsAg and HBeAg in cell culture supernatants were detected by ELISAs. **B** pHBV1.3 at different doses was transfected into HepG2 and Huh7 cells. After 48 h, the expression of GP73 was detected by qRT-PCR. **C**, **D** The pHBV1.3 overexpression plasmid (0.4 μg/ml) was transfected into primary human hepatocytes (PHHs). The expression of HBsAg and GP73 was detected by Western blots (**C**). The expression of GP73 was analyzed by qRT-PCR. **D** The graphs show the means ± SDs, n = 3. *P < 0.05, **P < 0.01, ***P < 0.001, compared with the control group

The abnormal expression of GP73 is also closely related to HCC development [11]. Huh7 cells and HepG2 cells were transfected with pHBV1.3 to identify whether HBV infection promotes the expression of GP73. After 48 h, the protein levels of HBeAg and HBsAg in cell culture supernatants were detected by ELISAs. With increasing amounts of transfected plasmids (pHBV1.3), the expression levels of HBeAg and HBsAg increased gradually (Fig. 2A). qRT-PCR showed that with increasing amounts of transfected pHBV1.3, the expression level of GP73 increased gradually (Fig. 2B). To further confirm whether HBV antigens activate GP73 expression, we analyzed the expression of GP73 by Western blot and qRT-PCR analysis of the PHHs transfected with pHBV1.3. When HBV genes were overexpressed in PHHs, GP73 expression was also increased (Fig. 2C, D).

**Fig. 3:**
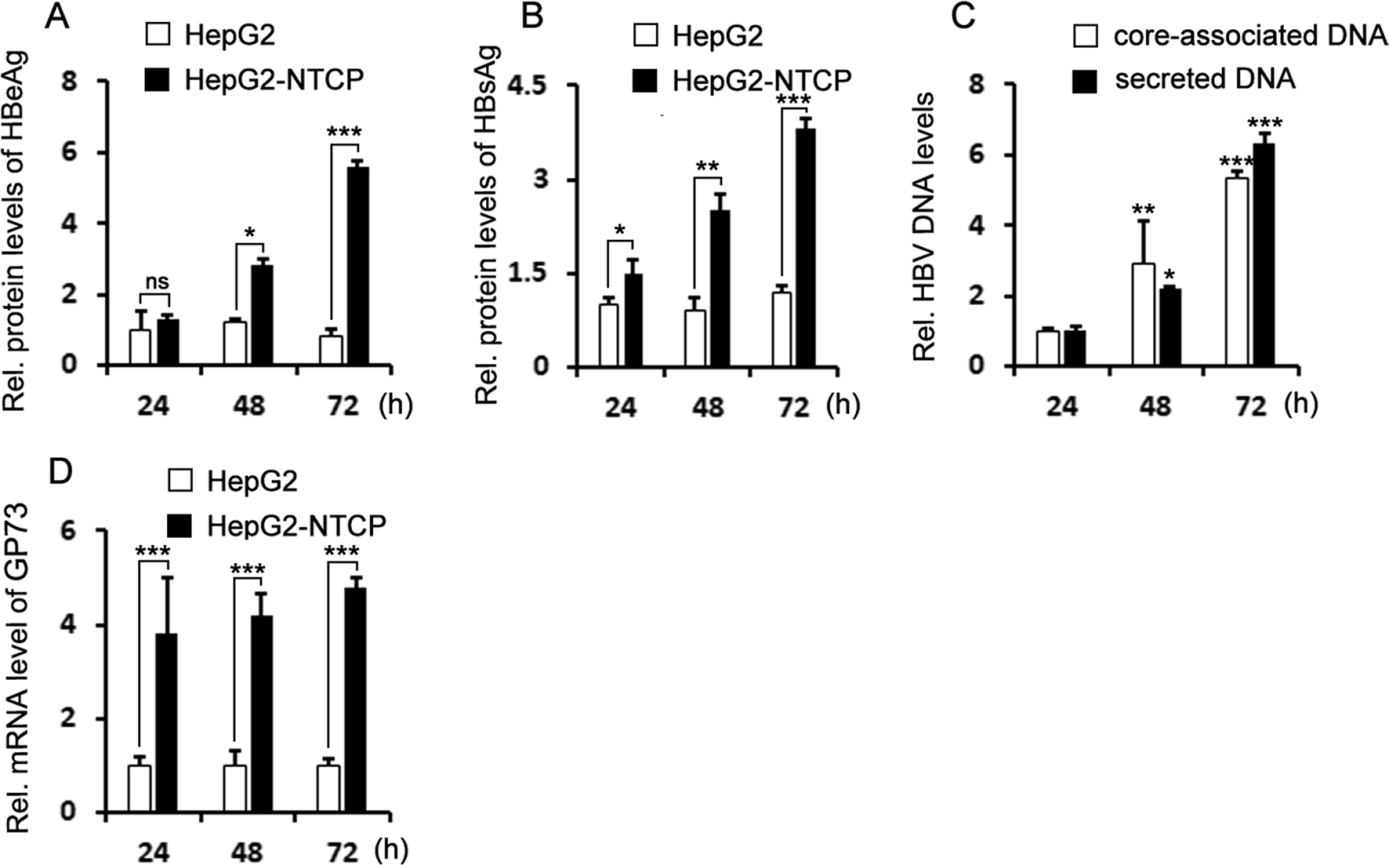
HBV induces the activation of GP73. HepG2 and HepG2-NTCP cells were infected with HBV at 1000 GE per cell. After 48 h, the expression of HBeAg (**A**) and HBsAg (**B**) in cell culture supernatants was detected by ELISAs; HBV genomic DNA (**C**) in HepG2-NTCP cells and cell culture supernatants was detected by qRT-PCR, and GP73 expression levels (**D**) were detected by qRT-PCR. The graphs show the means ± SDs, n = 3. *P < 0.05, **P < 0.01, ***P < 0.001, compared with the control group

Moreover, we investigated whether GP73 expression is activated by HBV infection by imitating natural infection of HBV in HepG2-NTCP cells. We first compared the HBV infection efficiency of HepG2-NTCP cells with that of HepG2 cells. When HepG2-NTCP cells and HepG2 cells were infected with HBV, HBeAg and HBsAg expression was upregulated in HepG2-NTCP cells but not in HepG2 cells (Fig. 3A, B). Further analysis of HBV core-associated DNA and secreted HBV core-associated DNA confirmed that HBV replication proceeded in HepG2-NTCP cells (Fig. 3C). Subsequently, we sought to investigate whether HBV infection could affect GP73 expression in the HepG2-NTCP system. When analyzing GP73 expression in the HBV-infected HepG2-NTCP cells, we found that the mRNA expression level of GP73 was upregulated (Fig. 3D).

Accordingly, the above results suggest that HBV induces the expression of GP73.

### GP73 activates CSC-related genes in L02 and HepG2 cells

**Fig. 4:**
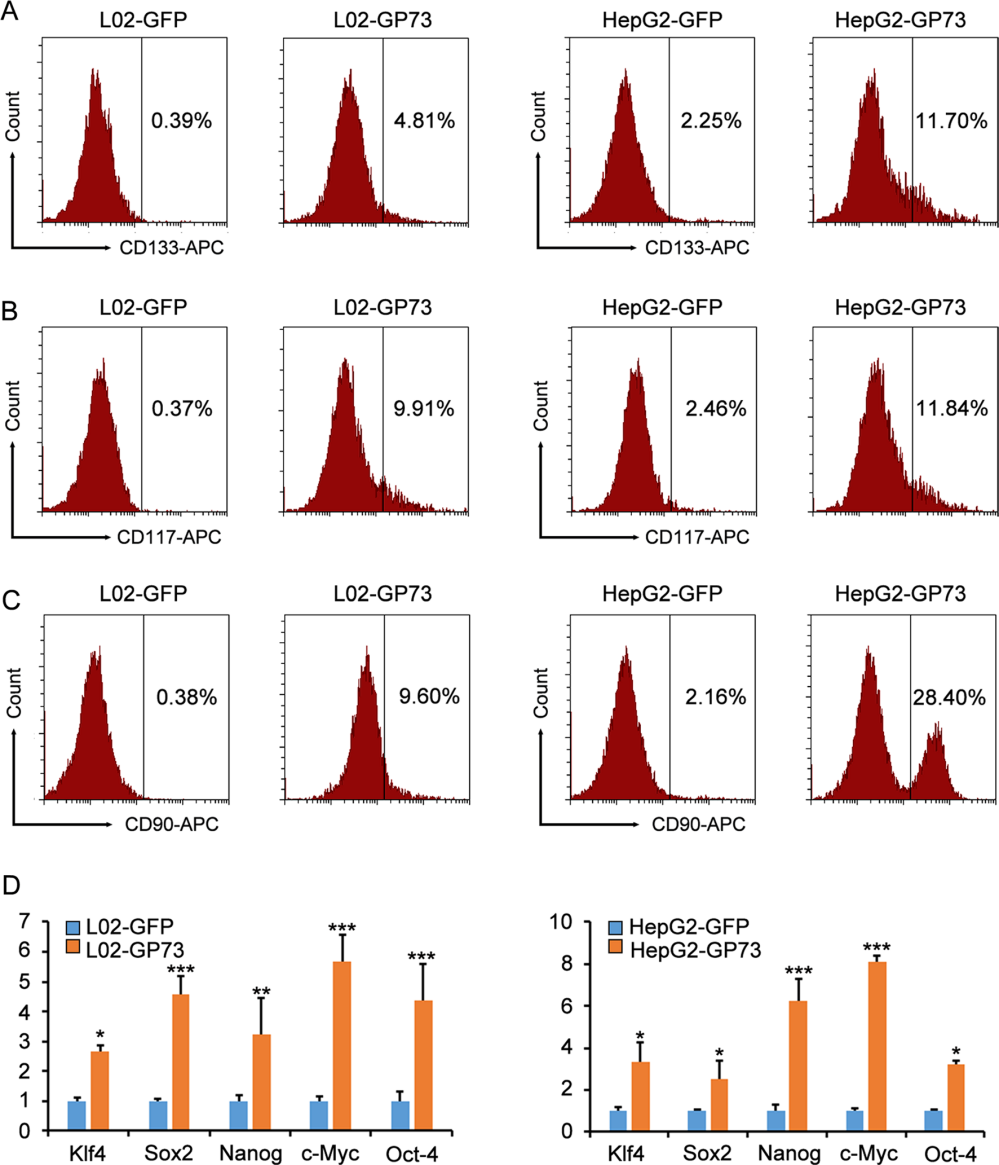
GP73 activates CSC-related genes in L02 and HepG2 cells. **A–C** Percentage changes in CD133^+^ (**A**), CD117^+^ (**B**), and CD90^+^ (**C**) cells in the L02 and HepG2 GP73-overexpressing cells were measured by flow cytometry. L02-GFP and HepG2-GFP cells were employed as controls compared with L02-GP73 and HepG2-GP73 cells. **D**, **E** The mRNA levels of Klf4, Sox2, Nanog, c-Myc, and Oct4 in the L02 and HepG2 GP73-overexpressing cells were determined by qRT-PCR. The graphs show the means ± SDs, n = 3. *P < 0.05, **P < 0.01, ***P < 0.001, compared with the control group

It has been reported that there is a close correlation between HCC and abnormal GP73 expression [6, 12]. However, direct evidence that GP73 induces hepatocarcinogenesis is still lacking. Here, we investigated the roles of GP73 in activating CSC-related markers in L02 cells and HepG2 cells. We generated 4 stable overexpression cell lines (L02-GFP, L02-GP73, HepG2-GFP, and HepG2-GP73) that stably expressed GFP and GP73. Then, the CSC surface markers (CD117, CD133, and CD90) of the 4 stable cell lines were analyzed. The results showed that the proportion of CD133^+^, CD117^+^, CD90^+^ cells increased to varying degrees (Fig. 4A–C). The qRT-PCR results showed that Sox2, Klf4, Nanog, c-Myc, and Oct4 were upregulated in HepG2 and L02 cells after GP73 overexpression (Fig. 4D). These results demonstrate that GP73 activates CSC-related genes in L02 and HepG2 cells.

### GP73 facilitates stemness in normal liver and hepatoma cells

**Fig. 5:**
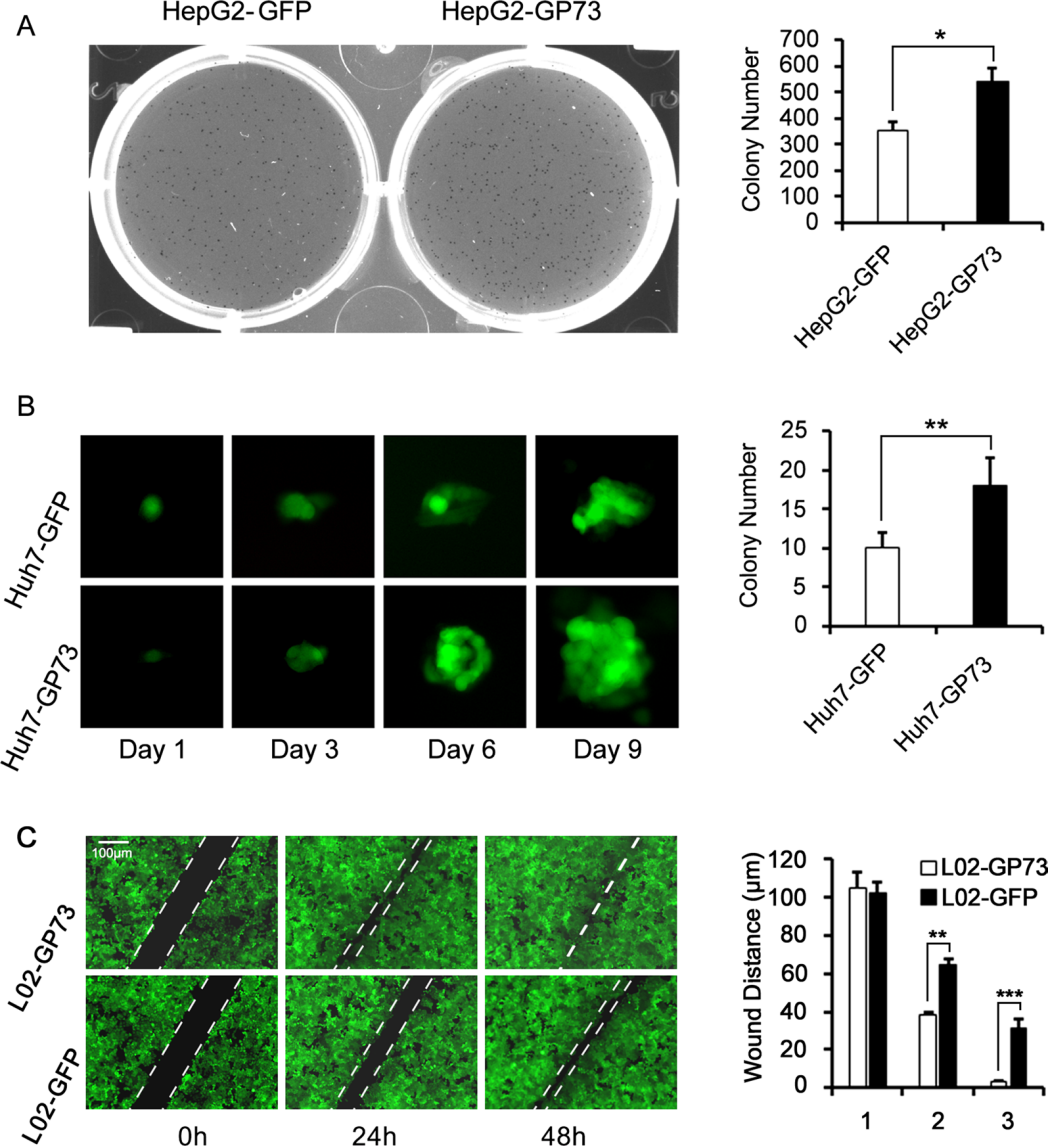
GP73 facilitates CSC generation in HepG2 and Huh7 cells. **A** HepG2-GFP and HepG2-GP73 cells were cultured in ultralow attachment 6-well plates at 2000 cells/well density. The sphere formation ability of the HepG2-GFP and HepG2-GP73 cells was investigated. **B** Huh7-GFP and Huh7-GP73 cells were sorted into ultralow attachment 96-well plates at a density of 1 cell/well by flow cytometry. The spheroid phenotype changes and numbers of the HepG2-GFP and HepG2-GP73 cells were examined by microscopy. **C** A wound-healing assay to test the migration of L02-GP73 and L02-GFP cells is shown (scale bar = 100 μm). The graphs show the means ± SDs, n = 3. *P < 0.05, **P < 0.01, compared with the control group

Because GP73 activates CSC-related genes in L02 and HepG2 cells, we further measured the sphere formation ability of HepG2-GP73 cells and Huh 7-GP73 cells and measured the migration of L02-GP73 cells. Sphere formation assays revealed that the colony number of the HepG2-GP73 cells was higher than that of the controls (Fig. 5A). In addition, the colony growth rate of the Huh7-GP73 cells was faster than that of the Huh7-GFP cells (Fig. 5B). These results suggest that GP73 enhances the colony-formation ability of HepG2 and Huh7 cells. Moreover, wound healing assays showed increased cell migration in L02-GP73 cells (Fig. 5C). The results above show that GP73 enhances stemness in normal liver and hepatoma cells.

### GP73 increases HepG2- and Huh7-mediated tumorigenesis in nude mice

**Fig. 6:**
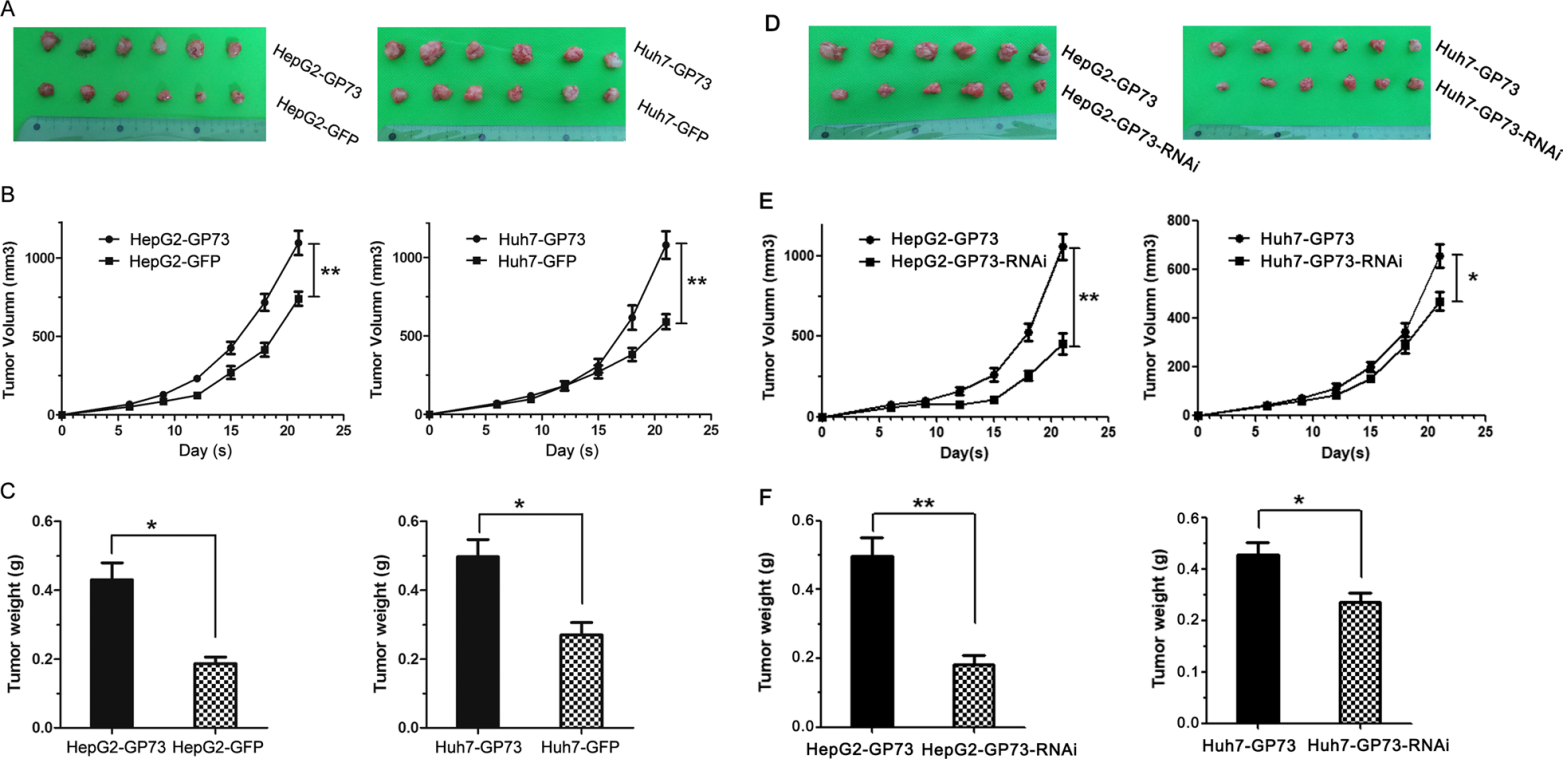
GP73 facilitates HepG2 and Huh7 tumorigenesis in nude mice. **A**, **D** The tumor images in each group are shown. **B**, **E** The average tumor volume in each group is shown. **C**, **F** The average tumor weight in each group is shown. The graphs show the means ± SDs, n = 6. *P < 0.05, **P < 0.01, compared with the control group

Because we found that the forced expression of GP73 enhanced stemness in Huh7 and HepG2 cells, the effect of GP73 overexpression on tumorigenesis in nude mice was further evaluated, and the results showed that tumorigenesis of the HepG2-GP73 and Huh7-GP73 cells increased compared with that of the controls (Fig. 6A, B). Furthermore, the average weight of the tumors was calculated. The average HepG2-GP73 and Huh7-GP73 tumor weights were significantly higher than those of the control tumors (Fig. 6C).

We further verified the effect of GP73 on the tumorigenic ability of HepG2 and Huh7 cells. We established stable shRNA-GP73 knockdown cell lines named Huh7-GP73-RNAi and HepG2-GP73-RNAi. Then, the effect of GP73 knockdown on tumorigenesis was investigated. The results showed that the tumorigenesis of the HepG2-GP73-RNAi and Huh7-GP73-RNAi cells was decreased compared with that of the HepG2-GP73 and Huh7-GP73 cells (Fig. 6D–F).

These results indicate that GP73 enhances the tumorigenicity of HepG2 and Huh7 cells in nude mice.

### HBV-induced GP73 expression represses the expression of NF-κB, IFN-λ1, IFN-β, TNF-α, and IL-6

**Fig. 7:**
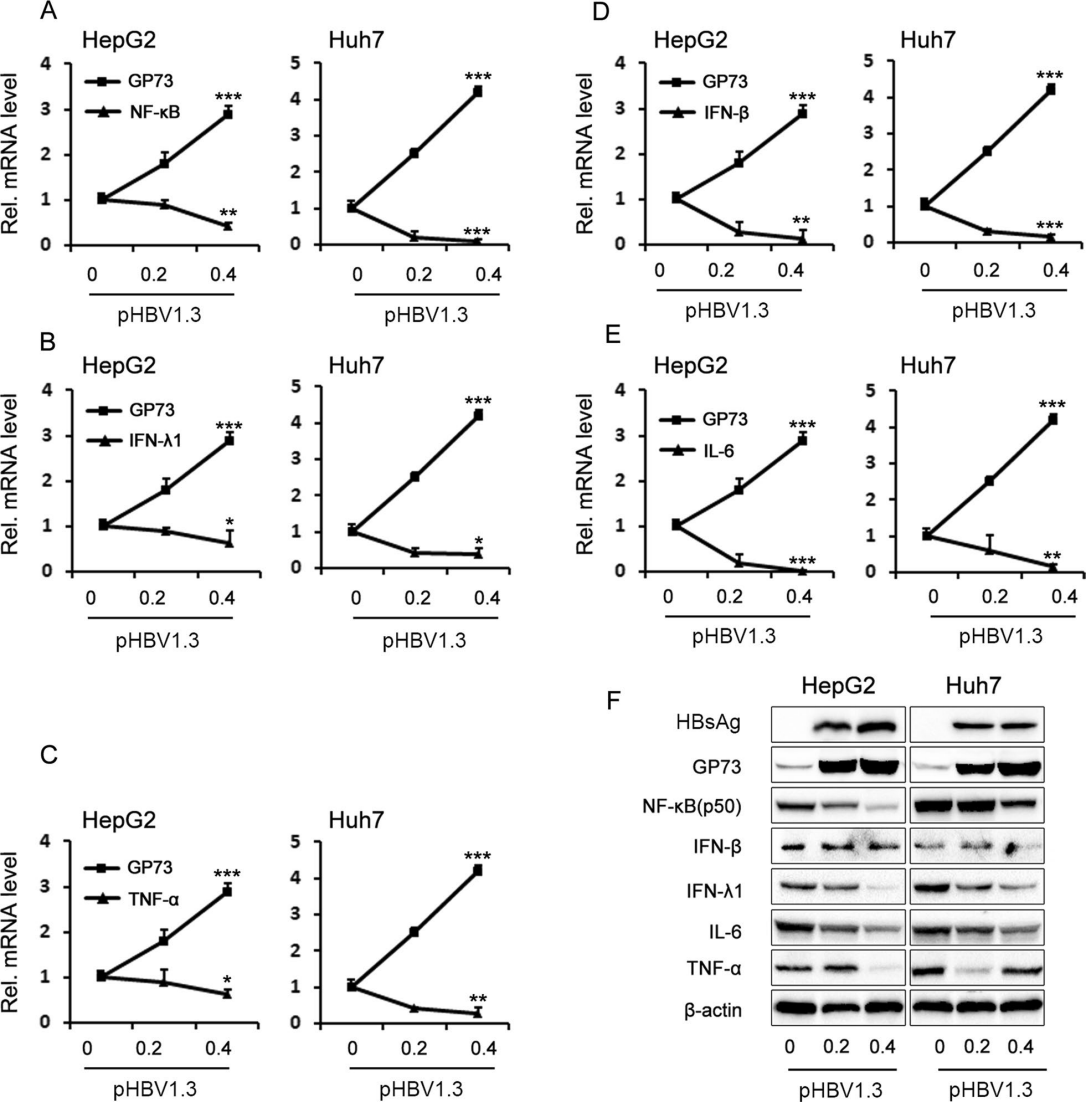
HBV induces GP73 expression to repress the expression of NF-κB, IFN-β, IFN-λ1, IL-6, and TNF-α. The plasmid pHBV1.3 was transfected into HepG2 and Huh7 cells at different doses. After 48 h, the expression levels of GP73, NF-κB, IFN-β, IFN-λ1, IL-6, and TNF-α were detected by qRT-PCR (**A–E**) and Western blotting (**F**). The graphs show the means ± SDs, n = 3. *P < 0.05, **P < 0.01, ***P < 0.001, compared with the control group

The above results indicate that HBV activates GP73 expression to promote HCC development. We next sought to clarify the molecular mechanism by which GP73 facilitates HCC development. Previous reports have shown that host innate immunity activates viral invasion and produces a series of antiviral cytokines [18]. We performed a series of experiments to assess whether the expression of GP73 regulates the host innate response. When analyzing GP73 and NF-κB in two hepatoma cell lines (HepG2 and Huh7) transfected with pHBV1.3, we found that GP73 mRNA levels positively correlated with pHBV1.3 expression, and NF-κB mRNA levels negatively correlated with pHBV1.3 expression (Fig. 7A). Further study also revealed that the forced expression of HBV genes significantly upregulated GP73, which downregulated the expression of IFN-λ1, IFN-β, TNF-α, and IL-6 in HepG2 and Huh7 cells at both the mRNA and protein levels (Fig. 7B–D).

### HBV facilitates HCC development by activating GP73 to repress the host innate immune response

**Fig. 8:**
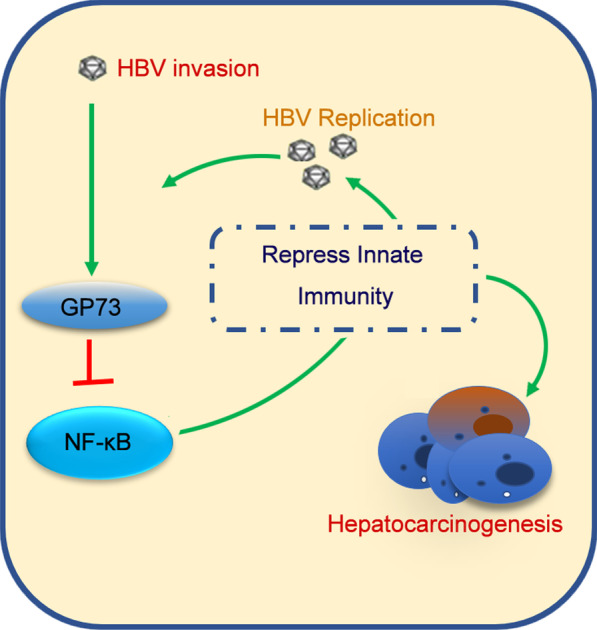
HBV facilitates HCC development by activating GP73 to repress the host's innate immune response. HBV induces GP73 overexpression in cells, inhibits NF-κB and its downstream signaling pathways, and inhibits the cellular innate immune response. The inhibition of the cellular innate immune response is conducive to HBV replication, leading to the loss of monitoring of abnormal hepatocytes, which eventually results in HBV-related hepatocarcinogenesis

Altogether, these results suggest that HBV promotes GP73 overexpression in cells and inhibits NF-κB and its downstream signaling pathways, resulting in the inhibition of the cellular innate immune response. The inhibition of the cellular innate immune response is conducive to HBV replication, leading to the loss of monitoring of abnormal hepatocytes, which eventually results in HBV-related hepatocarcinogenesis (Fig. 8).

## Discussion

Chronic HBV carriers are more likely to develop HCC than noncarriers [19–21]. HBV persistently replicates in hepatocytes without obvious cell damage or death, suggesting noncytopathic virus [22]. However, HBV-related HCC develops in an inflammatory environment caused by chronic HBV infection, indicating that the occurrence of HCC is immune-mediated [23]. Continuous HBV replication in hepatocytes leads to persistent inflammation and prolonged CLD, the leading cause of HCC [24]. During HBV infection, hepatocytes begin to express certain proteins abnormally. The activation of certain proteins that attenuate the host immune response after HBV infection may cause chronic infection.

Studies have shown that viral infection can activate the expression of GP73 in hepatocytes [8, 17]. We also found that HBV infection upregulates GP73 during hepatocarcinogenesis. The role of HBV in the activation of GP73 expression was further confirmed in HepG2-NTCP cells.

Previous reports have demonstrated that the concentration of serum GP73 is significantly upregulated in patients with CLD or HCC [12]. Although the relationship between clinical viral liver injury and abnormal expression of GP73 has been confirmed [10–12], the role of GP73 in the development of HBV-related HCC is still poorly studied. We showed that GP73 promotes HCC development by repressing the innate immune response. Flow cytometry and qRT-PCR analysis revealed that GP73 overexpression upregulated the expression of CSC-related genes in L02 and HepG2 cells. Sphere formation assays showed that GP73 enhances the colony-formation ability of HepG2 and Huh7 cells. Wound healing assays revealed that GP73 facilitates the migration of L02 cells. More importantly, xenograft mouse analysis indicated that GP73 enhanced the tumorigenicity of HepG2 and Huh7 cells in nude mice.

For chronic HBV infection, the greatest challenge for viral survival in vivo is successfully escaping immune-mediated elimination. During chronic HBV infection, varying degrees of liver inflammation persist. Chronic HBV infection downregulates the expression of interferon, and interferon therapy shows a strong antiviral effect in HBV transgenic mice [25]. The imbalance of cytokine secretion in hepatocytes or leukocytes further hinders the immune system from clearing the virus. Moreover, the HBx antigen of HBV inhibits TNF-α- and FAS-mediated apoptosis by activating NF‑κB [26]. The nuclear transcription factor NF-κB plays an important role in cellular innate immunity [27]. When potential pathogens invade a cell, NF-κB is activated and triggers the production of a series of antiviral cytokines (IFN-β, TNF-α) [28]. In addition, NF-κB plays a key role in hepatocyte survival by intrahepatic balancing and inhibiting cytotoxic reactive oxygen species (ROS) accumulation [29]. Thus, NF-κB is essential for maintaining hepatocyte homeostasis [30].

This study evaluated the effect of HBV and GP73 on tumorigenicity and innate immune regulation. We have previously proven that forced expression of the HBV gene induces HCC development [13]. We found that GP73 also facilitates HCC development and that HBV activates its expression. Further expression analysis showed that the expression of innate immune response-related factors (IFN-β, IFN-λ1, IL-6, TNF-α, and NF-κB) was inversely regulated by HBV in both HepG2 and Huh7 cells. Thus, our results indicate that HBV activates GP73 to inhibit cellular innate immunity, protecting HBV-infected cells from immune-mediated cytotoxic lysis and facilitating HCC development.

In conclusion, our results reveal that HBV facilitates HCC development by activating GP73 to repress the host's innate immune response. Initially, HBV induces GP73 expression. GP73 promotes HCC development by inhibiting the innate immune response through the NF-κB signaling pathway. Accordingly, HBV may co-opt GP73 to maintain persistent infection by repressing cellular innate immunity through attenuation of NF-κB activity, leading to the occurrence of HCC. This study provides a new perspective for exploring the pathogenesis of HBV-related HCC. The results also provide preclinical support for GP73 as a potential HCC prevention or treatment target.

## Supplementary Information

**Additional file 1:** Primer sequences for qRT-PCR.

## Data Availability

All data and materials were presented in the text. It is also available from the corresponding author on request.

## Abbreviations

HCC: Hepatocellular carcinoma
NF-κB: Nuclear factor kappa B
HBV: Hepatitis B virus
GP73: Golgi protein 73
PHHs: Primary human hepatocytes
CSCs: Cancer stem cells
WCL: Whole cell lysate
MOI: Multiplicity of infection
HBsAg: Hepatitis B surface antigen
HBeAg: Hepatitis B e antigen
