# Supplementary material for: Hepatitis B virus promotes hepatocellular carcinoma development by activating GP73 to repress the innate immune response
Source: Infect Agent Cancer. 2022 Oct 4;17:52. doi: 10.1186/s13027-022-00462-y (PMC9533540; doi:10.1186/s13027-022-00462-y)
Supplement: Supplementary file 1 — Additional file 1: Primer sequences for qRT-PCR. [file 13027_2022_462_MOESM1_ESM.docx]

Additional file 1 Table S1 Primers for qRT-PCR

| **Gene** | **Primers (5ʹ -3ʹ)** |
| --- | --- |
| *GP73* | Forward: CACAAGGGAAGGGAAACGTG  Reverse: CGAAGCCTCTTCCACCTACA |
| *Klf4* | Forward: ATTACCCATCCTTCCTGCCC  Reverse: CACGATCGTCTTCCCCTCTT |
| *Sox2* | Forward: AGCTCGCAGACCTACATGAA  Reverse: TGGAGTGGGAGGAAGAGGTA |
| *Nanog* | Forward: ACCCAGCTGTGTGTACTCAA  Reverse: CTGCGTCACACCATTGCTAT |
| *c-Myc* | Forward: ATTCTCTGCTCTCCTCGACG  Reverse: AGCCTGCCTCTTTTCCACA |
| *Oct4* | Forward: AGAACATGTGTAAGCTGCGG  Reverse: GGTTCGCTTTCTCTTTCGGG |
| *IFN-β* | Forward: CTGCAACCTTTCGAAGCCTT  Reverse: AAGCCTCCCATTCAATTGCC |
| *IFN-λ1* | Forward: GCTGGTGACTTTGGTGCTAG  Reverse: AAGACAGGAGAGCTGCAACT |
| *IL-6* | Forward: AGTCCTGATCCAGTTCCTGC  Reverse: CTACATTTGCCGAAGAGCCC |
| *TNF-α* | Forward: GTCAACCTCCTCTCTGCCAT  Reverse: CCAAAGTAGACCTGCCCAGA |
| *NF-κB* | Forward: AATGGTGGAGTCTGGGAAGG  Reverse: TCTGACGTTTCCTCTGCACT |
| *GAPDH* | Forward: GGAAGGTGAAGGTCGGAGTCAACGG  Reverse: CTCGCTCCTGGAAGATGGTGATGGG |
